# Differentiating people with schizophrenia from healthy controls in a developing Country: An evaluation of portable functional near infrared spectroscopy (fNIRS) as an adjunct diagnostic tool

**DOI:** 10.3389/fpsyt.2023.1061284

**Published:** 2023-01-26

**Authors:** Bach Xuan Tran, Tham Thi Nguyen, Laurent Boyer, Guillaume Fond, Pascal Auquier, Hao Si Anh Nguyen, Ha Thi Nhi Tran, Hung Manh Nguyen, Jongkwan Choi, Huong Thi Le, Carl A. Latkin, Kalpana Isabel Nathan, Syeda F. Husain, Roger S. McIntyre, Cyrus S. H. Ho, Melvyn W. B. Zhang, Roger C. M. Ho

**Affiliations:** ^1^Institute for Preventive Medicine and Public Health, Hanoi Medical University, Hanoi, Vietnam; ^2^EA 3279, CEReSS, Research Centre on Health Services and Quality of Life, Aix Marseille University, Marseille, France; ^3^Institute of Health Economics and Technology, Hanoi, Vietnam; ^4^Hanoi Department of Health, Hanoi, Vietnam; ^5^Mai Huong Daycare Psychiatric Hospital, Hanoi, Vietnam; ^6^OBELAB, Inc., Seoul, Republic of Korea; ^7^Bloomberg School of Public Health, Johns Hopkins University, Baltimore, MD, United States; ^8^Department of Psychiatry and Behavioral Sciences, Stanford University, Stanford, CA, United States; ^9^Department of Paediatrics, Yong Loo Lin School of Medicine, National University of Singapore, Singapore, Singapore; ^10^Mood Disorder Psychopharmacology Unit, University Health Network, University of Toronto, Toronto, ON, Canada; ^11^Institute of Medical Science, University of Toronto, Toronto, ON, Canada; ^12^Department of Psychiatry, University of Toronto, Toronto, ON, Canada; ^13^Department of Pharmacology, University of Toronto, Toronto, ON, Canada; ^14^Department of Psychological Medicine, Yong Loo Lin School of Medicine, National University of Singapore, Singapore, Singapore; ^15^Family Medicine and Primary Care, Lee Kong Chian School of Medicine, Nanyang Technological University Singapore, Singapore, Singapore; ^16^Institute for Health Innovation and Technology, National University of Singapore, Singapore, Singapore

**Keywords:** spectrometry, near-infrared, NIR spectroscopy, cortical hemodynamic response, schizophrenia, Vietnam

## Abstract

**Introduction:**

This study aimed to evaluate portable functional near-infrared spectroscopy (fNIRS) device as an adjunct diagnostic tool in Vietnam to assess hemodynamics when people with schizophrenia and healthy controls performed cognitive tasks.

**Methods:**

One hundred fifty-seven participants were divided into schizophrenia (*n* = 110) and healthy controls group (*n* = 47), which were recruited by match of age, and gender. Hemodynamic responses in the frontal cortex were monitored with a 48-channel portable device during the Stroop Color-Word Test (SCWT) and Verbal Fluency Test (VFT). General linear model compared the differences in oxyhemoglobin (HbO_2_) levels between the two groups. The Receiver Operating Characteristic (ROC) graph was generated for each neuroanatomical area.

**Results:**

People with schizophrenia did not show significant activation in the frontal lobe during the SCWT and VFT as compared to pre-task. During the VFT, the area under the ROC curve of the bilateral dorsolateral prefrontal cortex, bilateral orbitofrontal cortex, bilateral frontopolar prefrontal cortex, and bilateral ventrolateral prefrontal cortex were greater than 0.7 (p < 0.001). The area under the ROC curve (AUC) for the right orbitofrontal cortex was maximal during the VFT (AUC = 0.802, 95%CI = 0.731–0.872). The Youden’s index reached a peak (0.57) at the optimal cut-point value (HbO_2_ cutoff <0.209 μmol/ml for schizophrenia) in which the sensitivity was 85%; specificity was 72%; positive predictive value (PPV) was 0.88; negative predictive value (NPV) was 0.68 and correct classification rate was 76%.

**Discussion:**

Assessing hemodynamics during VFT by portable fNIRS offers the potential as an adjunct diagnostic tool for schizophrenia in developing countries.

## 1. Introduction

Schizophrenia is a common psychiatric disorder that is characterized by positive symptoms including auditory hallucinations, thought interference, and delusion as well as negative symptoms including avolition, anhedonia, and blunted effect. The 1-year and lifetime prevalence of schizophrenia are 1 and 1.4%, respectively (1). The current diagnosis of schizophrenia is mainly based on clinical interviews, and structured clinical assessment according to Diagnostic Statistical Manual (DSM) or International Classification of Disease (ICD) (2). There is a need to require neuroimaging biomarkers to improve the objectivity and accuracy rate of diagnosis. According to Kraguljac et al. (3), the goal of developing diagnostic biomarkers is to detect the presence of the disease state of schizophrenia and to establish objective schizophrenia signatures. According to a recent review, the potential neuroimaging biomarker devices for schizophrenia include reduction of the cortical gray matter volume, dopamine hyperactivity, and hippocampal hyperactivity (3). Such biomarkers can only be detected by magnetic resonance imaging (MRI) and positron emission tomography (PET). The average fees of MRI and PET scans are approximately 1,400 USD and 5,700 USD, respectively, and require special imaging facilities. These neuroimaging methods pose a challenge to developing countries. Furthermore, people staying in rural areas do not have access to MRI and PET scans. There is a requirement to develop, apply, and validate portable function neuroimaging as an adjunct diagnostic tool for schizophrenia in developing countries.

**FIGURE 1:**
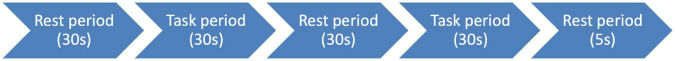
The paradigm of Stroop Color-Word Test (SCWT) adopted for Functional near-infrared spectroscopy (fNIRS).

Functional near-infrared spectroscopy (fNIRS) is a practical and cost-effective neuroimaging solution for developing countries. This device adopts a similar mechanism as functional MRI (fMRI) and utilizes infrared light to map brain activation based on assessing the changes in oxygenated, and deoxygenated hemoglobin, thus assessing the cerebral blood flow of each region (4). fNIRS was found to be significantly correlated with fMRI blood oxygen level-dependent (BOLD) signal regarding the respective neuroanatomical regions (5). This device also was found to have higher temporal resolution but lower spatial resolution than fMRI and PET (6). As compared to fMRI, the cost of fNIRS imaging is close to zero dollar per assessment, and the respondents can be measured in a sitting position rather than supine. The latest fNIRS device is portable (see Figure 1) and may be transported to rural areas. The validity of fNIRS to detect a psychiatric disorder and differentiate from other psychiatric disorders was investigated in major depressive disorders (7), bipolar disorders (8), and borderline personality disorders (9). For fNIRS research in schizophrenia, Kumar et al. (10) reported a compensatory hyperactivation in the right frontopolar cortex in those with schizophrenia, which may stem from the underlying deficits in working memory. As those with schizophrenia were found to have a reduced profile of executive functions compared to healthy controls (11), cognitive tasks assessing executive function may differentiate people suffering from schizophrenia and healthy controls utilizing the fNIRS scan (12). Verbal fluency test (VFT) (13, 14) and Stroop Color-Word Test (SCWT) (15) are common neuropsychological paradigms to differentiate people with schizophrenia from healthy controls under fNIRS. The VFT was validated in non-English speaking Asian populations including Japanese (16, 17), Taiwanese (18, 19), and Chinese (20). Differences in performances during the VFT and SCWT were able to differentiate people with schizophrenia from other psychiatric illnesses (e.g., bipolar disorder) (21, 22).

This study aimed to validate the portable fNIRS device modality as an adjunct diagnostic and distinguishing tool for people with schizophrenia from healthy controls. The null hypothesis was that there were no differences in oxyhemoglobin (HbO_2_) concentration between patients with schizophrenia and healthy controls during the VFT and SCWT.

## 2. Materials and methods

### 2.1. Study setting and procedure

A cross-sectional study was conducted from September 2020 to June 2022 at three medical facilities, including (1) Institute for Preventive Medicine and Public Health; (2) Mai Huong Daycare Psychiatric hospital; and (3) National Psychiatric Hospital No. 1. After providing the informed consent, participants were required to complete questionnaires on demographics, assessed by Positive and Negative Symptoms Scale (PANSS), and undergo the fNIRS scan.

### 2.2. Sample size and participants

One hundred fifty-seven people with schizophrenia (*n* = 110, Age = 42.3 ± 12.2 years, 39 females) and healthy controls (*n* = 47, Age = 40.2 ± 11.1 years, 17 females) were recruited for this study. The two groups of participants were matched by age, and gender. People with schizophrenia who are independent diagnosed by a qualified psychiatrist at the Mai Huong Daytime Psychiatric Hospital (40 patients), and National Psychiatric Hospital No1, Vietnam (70 patients) based on the ICD-10 diagnostic criteria (23). For healthy control group (47 participants), they were recruited from the community. After providing informed consent to participate in this study, they were invited to Institute for Preventive Medicine and Public Health, Hanoi Medical University to conduct a psychiatry screening test by the qualified psychiatrist. Particularly, the Brief Psychiatric Rating Scale (BPRS) was applied as a psychiatry screening test to recruit people into the healthy control group, and only people who were not suffering from any psychiatric disorders were recruited into this group and conducted fNIRS measurement.

Positive and negative symptoms of people with schizophrenia were evaluated by using the PANSS scale (24). The mean antipsychotic dose was calculated using olanzapine equivalence based on the World Health Organization’s Collaborative Center for Drug Statistics Methodology. All antipsychotic doses were converted to 1 mg olanzapine equivalent (25). Exclusion criteria included: (i) intellectual disability, (ii) chronic medical diseases (e.g., cardiovascular, respiratory, liver, and kidney diseases), (iii) neurological disorders (e.g., stroke, Parkinson’s disease, dementia), and (iv) substance use disorder. For healthy controls, they should not suffer from any psychiatric disorders.

### 2.3. Variables

Along with fNIRS measurement, information about demographic characteristics; and clinical characteristics (for patients with schizophrenia only) was also collected.

#### 2.3.1. Demographic characteristics included

Information about current age (years), gender (male/female), education level (secondary school or less/tertiary or higher) and family history of psychiatric disorders (yes/no).

#### 2.3.2. Clinical characteristics (for patient group only)

Included information such as age of illness (years), duration of illness (years), duration of untreated illness (months), pharmacotherapy, and PANSS score.

Positive and negative syndrome scale is considered the best-validated instrument for schizophrenia based on three domains: Positive, Negative, and General psychopathology (26). After the clinical interview with PANSS scale, the psychiatrist can assess the presence and severity of positive symptoms, negative symptoms, as well as general psychopathology among schizophrenia patients. This scale consisted of 30 items, with seven items regarding positive symptoms, seven items regarding negative symptoms, and sixteen items regarding general psychopathology symptoms. For each item, a seven-point Likert scale is utilized to assess the symptom severity (27). Finally, the total score of each domain is summed, with a higher score indicating a higher severity.

### 2.4. Computerized cognitive tasks

#### 2.4.1. The computerized version of SCWT

J. R. Stroop introduced the Stroop test, which has become a standard method for assessing executive function, processing speed, cognitive flexibility, and selective attention (28, 29). The Stroop task’s cognitive mechanism is related to selective attention, which requires the respondents to inhibit cognitive interference between ink color and word (30). Recently, the Stroop task has been used in several fNIRS studies, including studies for people with schizophrenia (22, 31, 32).

In this study, the E-prime software (Psychology Software Tools Inc.) displayed the Vietnamese computerized version of the SCWT and recorded the responses from participants. Five blocks comprised of three 30 s rest periods that alternated with two 30 s task periods (see Figure 1). Firstly, there were two rows of words that appeared in computer screen, with the words in the top row was a word in colored which was random in one of four colors, including “red,” “blue,” “yellow,” and “green.” The bottom row showed two different words and one of which was the color of the word in the top row. The respondents needed to determine the color of the word in the top row and selected the answer from the remaining row. Before performing the test, participants rested for 30 s. Then the participants performed the task for 30 s. The total runtime to complete the SCWT was 125 s.

#### 2.4.2. The verbal fluency test (VFT)

**FIGURE 2:**
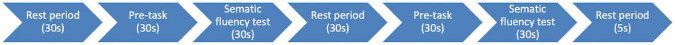
The paradigm for verbal fluency test (VFT) adopted for Functional near-infrared spectroscopy (fNIRS).

The VFT was used to evaluate executive function, lexical access speed, and vocabulary (33). The VFT has been chosen from among the many neuropsychological tests utilized to detect neurocognitive deficits in people with major psychiatric disorders (34, 35) because this is an executive test with distinct differences in performance and neuroimaging data among types of major psychiatric disorders (36, 37). To date, several studies have utilized VFT on fNIRS measurement to investigate the differences in cortical activation patterns between people with psychiatric disorders and healthy controls (35, 38–40). The Vietnamese version of VFT was applied in this study, and this task included two main parts. During the first part (i.e., pre-task), respondents were asked to say “A,” “B,” “C,” “D,” and “E” continuously for 30 s by Vietnamese. For the next part (i.e., the VFT task), the respondents had 30 s to list as many items as possible under a random category (see Figure 2). The semantic fluency of Vietnamese was assessed. The total runtime was 185 s.

### 2.5. The fNIRS measurement

**FIGURE 3:**
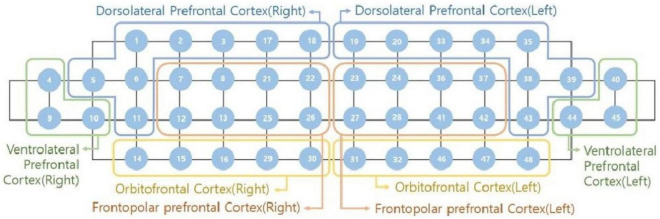
Location of 8 Brodmann neuroanatomical areas.

For the estimation of the HbO_2_ concentration, a portable fNIRS device (NIRSIT; OBELAB, Seoul, South Korea) with 48-channel and wavelengths 780 and 850 nm based on modified Beer–Lambert Law was utilized (41). This device included multi-distance source-detectors (24 dual-wavelength laser diodes and 32 photodetectors) that were separated by a distance of 1.5 cm. Forty-eight positions corresponding to 48 regions of the prefrontal cortex were examined by the source-detector pairs (Figure 3). To eliminate motion artifact and machine drift of physical contamination, high and low pass filters were used, with a frequency range of 0.005–0.1 Hz and a threshold of signal noise set at 30 dB (42). The average level of hemodynamic activation from −5 to 0 s before cognitive test were used as the baseline measurement. The response data were recorded 8.138 times per second. Finally, the average level of HbO2 was calculated as the mean level of HbO2 changes over the total runtime for each cognitive test. As Figure 3, the fNIRS device with the 48 channels were divided into eight subregions (43, 44), including the dorsolateral prefrontal cortex (right side and left side); the ventrolateral prefrontal cortex (right side and left side); the frontopolar prefrontal cortex (right side and left side); and the orbitofrontal cortex (right side and left side), and the mean HbO2 concentration in each Brodmann region was calculated according to the respective channels.

### 2.6. Data analysis and statistical analyses

Data analysis and visualization were performed using Stata 15.0 (Stata Corporation), and NIRSIT Analysis Toolbox v3.6.2. (OBELAB Inc.). Descriptive data were generated for healthy controls and people with schizophrenia. To compare the proportion between two groups of participants, the Chi-square and Fisher’s exact test were utilized. Differences in age between the healthy controls and people with schizophrenia were assessed by using the Wilcoxon rank-sum test (non-parametric method). The HbO_2_ levels between the rest period and task period were compared using the general linear model one level (GLM 1-Level) for healthy controls and people with schizophrenia. To compare the mean differences of HbO_2_ between the two groups, the general linear model two levels (GLM 2-Level) was utilized. The criterion for statistical significance was set at *p* < 0.05. We set contrast parameters between two among two groups, respectively, (healthy control vs. schizophrenia group). The one-way analysis of covariance (ANCOVA) was employed to compare mean HbO2 across these groups after adjusting for age, gender, and education.

In this study, the value of sensitivity, specificity, positive predictive value (PPV), and negative predictive value (NPV) were reported. Furthermore, the Receiver Operating Characteristic (ROC) curve and the area under the curve (AUC) are considered effective measures of accuracy has been explored with significant interpretations (45). This curve plays an important role in assessing the diagnostic ability of tests to distinguish the true state of individuals, determining the optimal cut-off values, and comparing two different diagnostic tests when they are performed on the same subject (45–47). In particular, ROC graphs were created based on the sensitivity and 1-specificity for all possible thresholds. In terms of AUC, rather than relying on a specific operational point, AUC summarizes the full position of the ROC curve (45, 48). The AUC is a useful and integrated measure of sensitivity and specificity that indicates the diagnostic test’s inherent validity (37). Sensitivity and specificity were transformed to Youden’s index (sensitivity + specificity–1) (49). Besides, AUC, PPV, and NPP, we selected the optimal cut-off point that maximized Youden’s index.

## 3. Results

The demographic characteristics and some clinical characteristics of the respondents are showed in Table 1. No significant differences between people with schizophrenia and healthy controls in terms of age (*p* = 0.177), and gender (*p* = 0.932). The majority of participants was male (65.5%, for healthy control group, and 64.4% for people with schizophrenia) were found. Most people with schizophrenia reported that they did not have any family psychiatric history with 90.0%. The mean age of healthy controls was 40.2 ± 11.1 years, while the mean age for schizophrenia was 42.3 ± 12.2 years. For people with schizophrenia, the mean age of onset of illness, duration of treated illness and duration of untreated illness were 27.6 ± 8.2 years, 17.6 ± 9.8 years, and 9.7 ± 25.9 months, respectively. The mean PANSS score was 79.8 ± 22.4, and the mean olanzapine equivalent dose for antipsychotic medications was 13.0 ± 7.1 mg.

**TABLE 1:** Characteristics of participants (*n* = 157).

Characteristics	Healthy controls		People with schizophrenia		*p*-value
	***n***	**%**	***n***	**%**	
**Total**	47	100.0	110	100.0	
**Gender**					
Male	30	63.8	71	64.6	0.932
Female	17	36.2	39	35.5	
**Highest education level**					
Secondary school or less (grade < 12)	3	6.4	47	42.7	<0.001
High school or higher (grade ≥ 12)	44	93.6	63	57.3	
**Family psychiatric history**					
No	47	100.0	99	90.0	0.025
Yes	0	0	11	10.0	
	**Mean**	***SD***	***Mean***	***SD***	
Age	40.2	11.1	42.3	12.2	0.177
Age at illness onset (years)	-	-	27.6	8.2	
Duration of treated illness (years)	-	-	17.6	9.8	
Duration of untreated illness (months)	-	-	9.7	25.9	
Olanzapine dose equivalent	-	-	13.0	7.1	
**Positive and negative syndrome scale (PANSS)**					
Positive			18.6	5.8	
Negative			19.4	6.3	
General			41.6	12.1	
Total score of PANSS			79.8	22.4	

**FIGURE 4:**
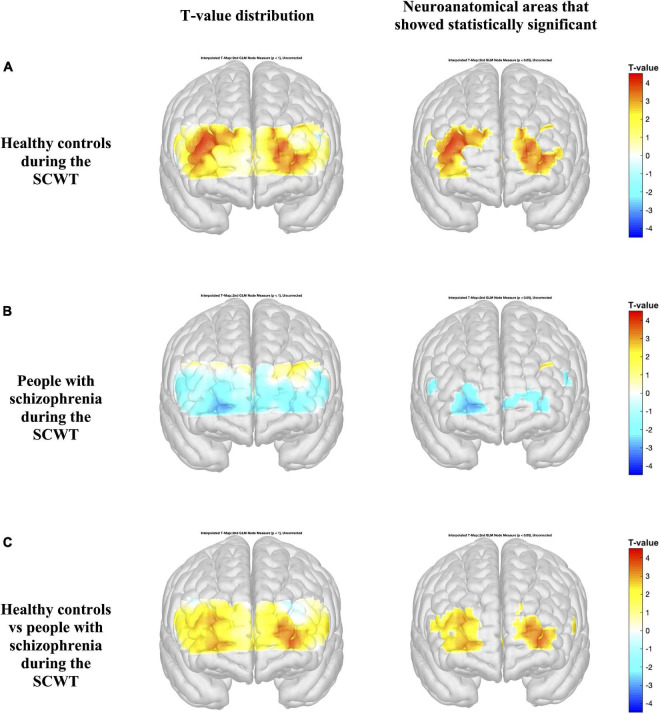
Activation maps based on the general linear model (GLM) compare the HbO_2_ levels during the rest period and Stroop Color-Word Test (SCWT) **(A,B)**. Differences in HbO_2_ levels during the SCWT are represented by colormap **(C)**. Figures on the left indicate the *T*-value distribution and figures on the right highlight the neuroanatomical areas that show statistical significance with *p* < 0.05 (*T*-value > 1.96).

**FIGURE 5:**
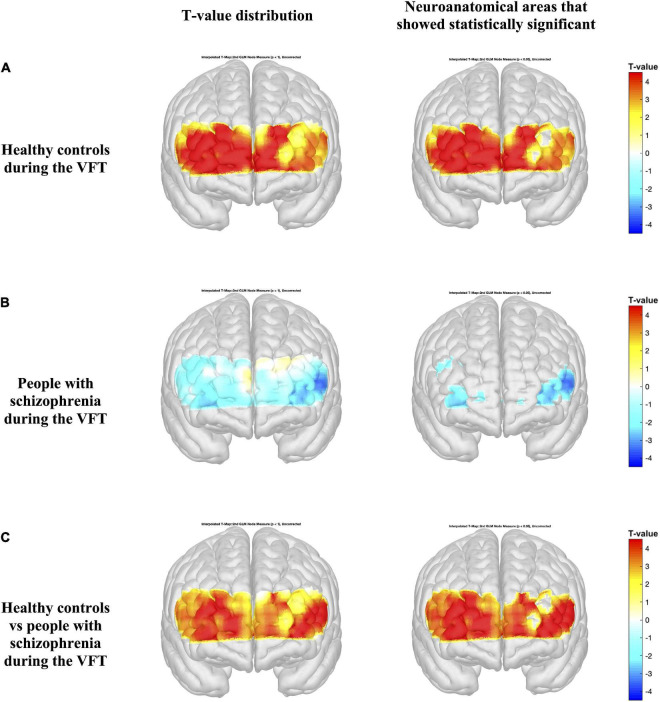
Activation maps from the general linear model (GLM) compare the HbO_2_ levels during the rest period and verbal fluency test (VFT) period **(A,B)**. Differences in HbO_2_ levels during the VFT are represented by colormap **(C)**. Figures on the left indicate the *T*-value distribution and figures on the right highlights the neuroanatomical areas that show statistical significance with *p* < 0.05 (*T*-value > 1.96).

Figures 4, 5 present the activation maps of people with schizophrenia and healthy controls during the SCWT and VFT, respectively, [left side figure highlights the *T*-value distribution and right side figure indicates the neuroanatomical regions that show statistical significance with *p* < 0.05 (*T*-value > 1.96)]. People with schizophrenia did not show significant activation during the SCWT and VFT (*p* > 0.05). In contrast, health controls group showed a higher significantly activation during two cognitive tasks. In addition, the color map showed significant differences in HbO2 in the most regions of prefrontal brain region between people with schizophrenia and healthy controls during SCWT and VFT (*p* < 0.05).

Table 2 shows that healthy controls had significantly higher level of HbO_2_ change in the some regions of interest of the prefrontal cortex during the SCWT. The highest level of HbO2 in healthy controls was recorded at 0.64 ± 1.58 in the left ventrolateral prefrontal cortex, while the lowest level of HbO2 concentration of people with schizophrenia was −0.24 ± 1.34 at the right orbitofrontal cortex. Furthermore, the difference in the level of HbO_2_ concentration in the left ventrolateral prefrontal cortex (*t* = 2.32, *p* < 0.05) and left dorsolateral prefrontal cortex (*t* = 2.49, *p* < 0.05) between two groups was statistically significant.

**TABLE 2:** The oxy-hemoglobin (Hbo2) levels changes between rest and task period in eight regions of interest of the prefrontal cortex during the Stroop Color-Word Test (SCWT) (mmol.mm) (*n* = 157).

Regions of interest	HC (Mean ± SD)	Schizophrenia (Mean ± SD)	*t*-value
**Right side**			
Ventrolateral prefrontal cortex	0.35 ± 1.35	0.01 ± 1.85	1.32
Dorsolateral prefrontal cortex	0.25 ± 0.66	0.03 ± 0.99	0.84
Frontopolar prefrontal cortex	0.34 ± 0.69	−0.04 ± 0.91	1.88
Orbitofrontal cortex	0.33 ± 0.70	−0.24 ± 1.34	1.87
**Left side**			
Ventrolateral prefrontal cortex	0.64 ± 1.58	−0.14 ± 1.12	2.32*
Dorsolateral prefrontal cortex	0.27 ± 0.57	−0.07 ± 1.14	2.49*
Frontopolar prefrontal cortex	0.29 ± 0.91	−0.04 ± 1.21	0.63
Orbitofrontal cortex	0.21 ± 1.17	−0.16 ± 2.67	0.34

(*p < 0.05; HC, Health controls).

Table 3 shows that healthy controls had significantly higher level of HbO_2_ change in all regions of interest of the prefrontal cortex during the VFT with *p* < 0.05. Particularly, the highest level of Oxy-Hb concentration of healthy controls was recorded at 1.24 ± 1.29 in the left of orbitofrontal cortex, followed by the left ventrolateral prefrontal cortex (1.05 ± 1.1.93), and right ventrolateral prefrontal cortex (0.98 ± 1.63). For people with schizophrenia, the lowest level of Oxy-Hb was −0.54 ± 1.70 in the left ventrolateral prefrontal cortex, and −0.54 ± 1.78 in the right orbitofrontal cortex.

**TABLE 3:** The oxy-hemoglobin (Hbo2) levels changes between rest and task period in eight regions of interest of the prefrontal cortex during the verbal fluency test (VFT) (mmol.mm) (*n* = 157).

Regions of interest	HC (Mean ± SD)	Schizophrenia (Mean ± SD)	*t*-value
**Right side**			
Ventrolateral prefrontal cortex	0.98 ± 1.63	−0.33 ± 2.18	2.66*
Dorsolateral prefrontal cortex	0.78 ± 1.11	−0.15 ± 1.47	2.99*
Frontopolar prefrontal cortex	0.79 ± 0.85	−0.01 ± 0.97	3.85*
Orbitofrontal cortex	0.80 ± 0.84	−0.54 ± 1.78	2.60*
**Left side**			
Ventrolateral prefrontal cortex	1.05 ± 1.93	−0.54 ± 1.70	3.48*
Dorsolateral prefrontal cortex	0.55 ± 0.94	−0.14 ± 1.21	3.06*
Frontopolar prefrontal cortex	0.79 ± 1.50	−0.32 ± 1.16	3.91*
Orbitofrontal cortex	1.24 ± 1.29	−0.49 ± 2.43	2.92*

(*p < 0.05; HC, Health controls).

**FIGURE 6:**
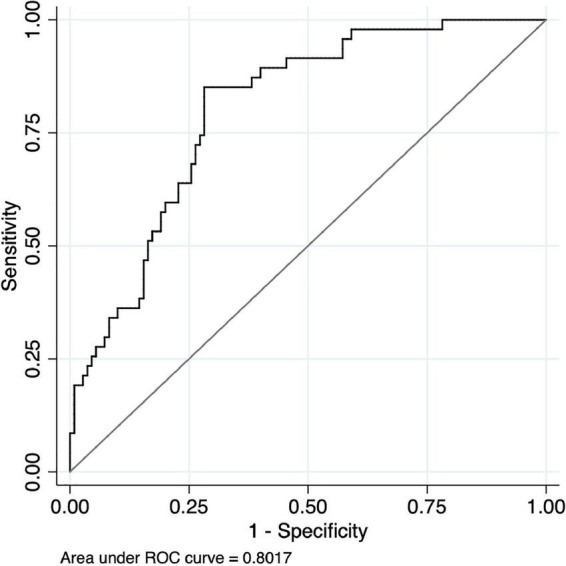
Receiver Operating Characteristic (ROC) curve of hemodynamic responses on the right of orbitofrontal cortex between healthy controls and people with schizophrenia during the verbal fluency test.

The area under the ROC curve (AUC) and asymptotic normal (95%CI) was estimated for each region during the SWCT and VFT (see Figure 6 and Tables 4, 5). The value of AUC ranged from 0.575 to 0.657 during the SCWT and from 0.717 to 0.802 during the VFT. During the SCWT, the highest value of area under the ROC curve was recorded in the right orbitofrontal cortex with AUC = 0.657 (95%CI = 0.568–0.746), while the opposite finding was observed for the right ventrolateral prefrontal cortex with AUC = 0.562 (95%CI = 0.473–0.650). During the VFT, the AUC was greater than 0.7 (*p* < 0.001) in all regions of interest. In which, the highest level of AUC during VFT was 0.802 (AUC = 0.802, 95%CI = 0.731–0.872) in the right orbitofrontal cortex, followed by the right frontopolar prefrontal cortex (AUC = 0.751, 95%CI = 0.674–0.828) and left ventrolateral prefrontal cortex (AUC = 0.751, 95%CI = 0.666; 0.835).

**TABLE 4:** Performance of predicting the diagnosis of schizophrenia by portable functional near-infrared spectroscopy (fNIRS) during the Stroop Color-Word Test.

Region of interest	Right side			Left side		
	**AUC**	**95% CI**	***p*** **-value**	**AUC**	**95% CI**	***p*** **-value**
Ventrolateral prefrontal cortex	0.558	0.466–0.650	0.217	0.646	0.553–0.737	0.002
Dorsolateral prefrontal cortex	0.600	0.502–0.692	0.046	0.606	0.514–0.700	0.023
Frontopolar prefrontal cortex	0.655	0.565–0.745	<0.001	0.575	0.477–0.672	0.133
Orbitofrontal cortex	0.657	0.568–0.746	<0.001	0.582	0.491–0.672	0.077

AUC, area under receiver operating characteristic curve.

**TABLE 5:** Performance of predicting the diagnosis of schizophrenia by portable functional near-infrared spectroscopy (fNIRS) during the verbal fluency test (VFT).

Region of interest	Right side			Left side		
	**AUC**	**95% CI**	***p*** **-value**	**AUC**	**95% CI**	***p*** **-value**
Ventrolateral prefrontal cortex	0.717	0.634– 0.800	<0.001	0.751	0.666–0.835	<0.001
Dorsolateral prefrontal cortex	0.740	0.656–0.823	<0.001	0.725	0.636–0.814	<0.001
Frontopolar prefrontal cortex	0.751	0.674–0.828	<0.001	0.724	0.632–0.815	<0.001
Orbitofrontal cortex	0.802	0.731–0.872	<0.001	0.747	0.672–0.821	<0.001

AUC, area under receiver operating characteristic curve.

For the region with highest the area under the ROC curve (right orbitofrontal cortex when performing VFT), the value of sensitivity, specificity, PPV, NPV, and optimal HbO_2_ cut-off point was reported. In particularly, the highest level of Youden’s index was reached at 0.57 with the optimal cut-point of level HbO2 value 0.209 (HbO_2_ cut-off ≥0.209 μmol/ml for healthy controls; HbO_2_ cut-off <0.209 for people with schizophrenia) in which the sensitivity was 85%; specificity was 72%; PPV was 0.88; NPV was 0.68, and classification accuracy was 76% (Table 6).

**TABLE 6:** The sensitivity, specificity, positive predictive, and negative predictive values (NPVs) of the HbO_2_ cut-off point for differentiating healthy controls from schizophrenia patients at the right orbitofrontal cortex during the verbal fluency test (VFT).

HbO_2_ cut-off point (Unit: μ M)	Sensitivity	Specificity	Positive predictive value	Negative predictive value	Correctly classified	Youden’s index
0.088	0.85	0.64	0.85	0.65	0.70	0.49
0.102	0.85	0.65	0.85	0.65	0.71	0.50
0.112	0.85	0.65	0.85	0.66	0.71	0.51
0.121	0.85	0.66	0.85	0.66	0.72	0.51
0.130	0.85	0.67	0.86	0.67	0.73	0.52
0.136	0.85	0.68	0.86	0.67	0.73	0.53
0.155	0.85	0.69	0.86	0.67	0.74	0.54
0.169	0.85	0.70	0.87	0.67	0.75	0.55
0.208	0.85	0.71	0.87	0.67	0.75	0.56
0.209*	0.85	0.72	0.88	0.68	0.76	0.57
0.212	0.83	0.72	0.88	0.64	0.75	0.55
0.213	0.81	0.72	0.87	0.62	0.75	0.53
0.250	0.79	0.72	0.87	0.60	0.74	0.51
0.265	0.77	0.72	0.87	0.57	0.73	0.48
0.280	0.74	0.72	0.86	0.55	0.73	0.46
0.291	0.74	0.73	0.86	0.55	0.73	0.47
0.300	0.72	0.73	0.86	0.53	0.73	0.45
0.313	0.72	0.74	0.87	0.54	0.73	0.46
0.317	0.70	0.74	0.87	0.51	0.73	0.44

*Optimal cut-off value of oxy-hemoglobin, >Optimal cut-off suggests healthy control; <Optimal cut-off suggests the diagnosis of schizophrenia.

## 4. Discussion

We found that fNIRS combined with neuropsychological tests succeeds in differentiating people with schizophrenia from healthy controls. The best schizophrenia diagnosis prediction was found for the combination of VFT and fNIRS with a focus on the right orbitofrontal cortex and a cutoff <0.209 μmol/ml for schizophrenia (sensitivity was 85%; specificity was 72%; PPV was 0.88; NPV was 0.68 and classification accuracy was 76%). Our positive predictive value (88%) is comparable with previous fNIRS studies to differentiate schizophrenia from healthy controls (84.7–89.7%) (13, 14, 50–53). Our findings are in line with previous fNIRS research that found the prefrontal cortex, a key neuroanatomical area that demonstrates pathology in important psychiatric illnesses including schizophrenia (54, 55), major depressive disorder (56), bipolar disorder (8), generalized anxiety disorder (57), and borderline personality disorder (9). Furthermore, underdevelopment in the right orbitofrontal cortex may underlie vulnerability to psychosis and the core symptoms of schizophrenia (58). Particularly, the studies by Chou et al. (53) and Yang et al. (13) have indicated that the classification accuracy of fNIRS devices in distinguishing schizophrenia from healthy controls ranged from 68.2 to 79.7% and 66.5 to 85.0%, respectively. These differences in the classification accuracy of fNIRS devices might result from variations in the VFT’s design. While the Japanese VFT or Chinese utilize syllabary, we chose Vietnamese alphabets. Actually, the lexical retrieval strategies of alphabetic and non-alphabetic languages differ (59). Therefore, it can affect cortical activity when performing this test. For example, at least two variations of Chinese VFT have been developed in fNIRS studies on people with schizophrenia, because they use different syllable systems (60). Specifically, the VFT developed by Li et al. (55) is applied to people in mainland China and differed from that used by Yang et al. (13) for residents in Taiwan.

Executive function is a group of complex cognitive activities (e.g., working memory and impulse controls) that contributes to organizing, planning, and making decisions before conducting tasks. Executive impairment is one of the core pathological traits of schizophrenia. The VFT evaluates word production, executive abilities, as well as cognitive flexibility (61). Before the development of fNIRS, the VFT was used to study cortical activation pattern of people with schizophrenia by functional magnetic resonance imaging (fMRI) (62). In this study, semantic fluency of Vietnamese language was assessed instead of phonemic fluency. Previous study found that schizophrenia was found to be associated with more compromises to the semantic fluency as compared to phonemic fluency (63). A recent meta-analysis found that the fNIRS-VFT paradigm enhances understanding, detection and differentiating various psychiatric conditions, and has the potential for developing cost-effective neuroimaging biomarkers for clinical psychiatry (64). Hence, it is not surprising that the VFT is a good neuropsychological test to distinguish hemodynamic activity between patients with schizophrenia and healthy controls by fNIRS measuring. A notable finding in the current study is the differences in HbO2 concentration between healthy group and schizophrenia group during the VFT, and this difference may be caused by underlying biological etiology and neuropathology. Our findings are consistent with previous functional neuroimaging studies. A fMRI study found that a specific medio-prefronto-striato-thalamic functional dysconnectivity detected by VFT and implicated as the pathophysiology of schizophrenia (65). Furthermore, sigma non-opioid intracellular receptor 1 (SIGMAR1) gene polymorphism is involved in the pathogenesis of schizophrenia (66). People with schizophrenia who are Pro carriers for SIGMAR1 gene have significantly lower activation of the right pre-frontal cortex during the VFT as compared to healthy people who are homozygous for the Gln/Gln genotype (66).

Further fNIRS research is needed to evaluate other cognitive tests such as Wisconsin Card Sorting Test and working memory task (e.g., N-back), which may be helpful in differentiating people with schizophrenia and healthy controls (67). fNIRS can be an objective tool to assess changes in brain hemodynamics after computer-based cognitive training in addition to improvement of scores in neuropsychological tests (68). A meta-analysis found that second generation antipsychotics such as clozapine, olanzapine, quetiapine, and risperidone produce a mild remediation of cognitive deficits in schizophrenia (69). fNIRS can be applied and monitored the changes in brain hemodynamics after the initiation of antipsychotic treatment. Portable fNIRS scan may have the potential to assess cognitive function in people who are at ultra-high risk for schizophreniform psychosis and first-episode schizophrenia (70). A longitudinal study is required to assess the capability of portable fNIRS in predicting the diagnosis of schizophrenia in young people who present with early psychosis.

To improve classification accuracy of fNIRS device, future diagnostic methods should incorporate fNIRS and other biomarkers such as interleukin (IL)-8 and superoxide dismutase which were found to correlate with executive function in people with schizophrenia (71). As performances in VFT could differentiate people with the first episode of schizophrenia and healthy controls (72), monitoring hemodynamics when performing VFT by the portable fNIRS scan could enhance the accuracy of diagnosis of the first episode of schizophrenia and allows early intervention.

This study has several limitations that should be considered when interpreting the finding, especially regarding the duration of illness, gender and clinical stage, and medication (73–76). Firstly, this is a cross-sectional study and a longitudinal study is needed to follow the changes in HbO2 concentration during the course of illness as well as the impact of antipsychotic medications on hemodynamic response when performing the cognitive tests. Secondly, fNIRS device in this study mainly assesses cortical regions and could not measure the changes of hemodynamics level of subcortical structures such as the cerebellum. Thirdly, because of the small sample, this study was not able to assess the hemodynamic activation of different types of schizophrenia as well as other characteristics such as age, gender and clinical stage of schizophrenia. Furthermore, this study lack results on behavioral performance during cognitive tests, and further study is required to explore this information for Vietnamese schizophrenia patients.

## Conclusion

In conclusion, this study indicates that during the VFT, the right orbitofrontal cortex is the most important neuroanatomical region to distinguish people with schizophrenia from healthy controls. The portable fNIRS device may be considered an adjunct diagnostic tool for schizophrenia.

## Data availability statement

The raw data supporting the conclusions of this article will be made available by the authors, without undue reservation.

## Ethics statement

The studies involving human participants were reviewed and approved by the Vietnam Ministry of Health under Decision No 850/QD-BYT. The protocol of this study was approved by the Institutional Review Board of Hanoi Medical University (Number 58/GCN-HDDNCYSH-DHYHN). The patients/participants provided their written informed consent to participate in this study.

## Author contributions

BT, HT, HL, CL, KN, RM, CH, and RH: conceptualization. TN, HT, HuN, and HaN: data curation. BT, TN, HaN, LB, GF, PA, SH, JC, RM, CH, CL, RH, and MZ: formal analysis. BT, TN, HT, RM, SH, JC, CH, CL, RH, and MZ: methodology. BT, TN, HaN, JC, and RH: software. BT, LB, GF, PA, HL, HT, HaN, RM, CH, CL, RH, and MZ: supervision. TN, HaN, HuN, and HT: investigation. BT, LB, GF, PA, HL, JC, RH, and MZ: writing—original draft. All authors contributed to the article, writing—review and editing, and approved the submitted version.
